# C-sections and hospital characteristics: a long term analysis on low-risk deliveries

**DOI:** 10.1007/s43999-022-00014-2

**Published:** 2022-12-14

**Authors:** Inês Joaquim, Luís Nobre Pereira, Carla Nunes, Céu Mateus

**Affiliations:** 1 Escola Nacional de Saúde Pública, Universidade Nova de Lisboa, Avenida Padre Cruz, 1600-560 Lisboa, Portugal; 2 Research Centre for Spatial and Organizational Dynamics (CIEO) - University of Algarve, School of Management, Hospitality and Tourism, University of Algarve, Campus da Penha, 8005-139 Faro, Portugal; 3 Centro de Investigação em Saúde Pública, Escola Nacional de Saúde Pública, Universidade Nova de Lisboa, Avenida Padre Cruz, 1600-560 Lisboa, Portugal; 4 Division of Health Research, HI One, Lancaster University, Sir John Fisher Drive, Lancaster, LA1 4AT UK

**Keywords:** Caesarean sections, Hospital resources, Low-risk, Fractional response models

## Abstract

**Background:**

Policymakers aim to reduce C-section (CS) rates, due to well documented overtreatment. However, little is known about how hospital characteristics relate to their c-section rates on low-risk deliveries (CSR-LRD).

**Methods:**

CSR-LRD were computed using inpatient data from all Portuguese National Health Service hospitals (2002-2011). Linear and Fractional Response Models were estimated to quantify the relationship between CSR-LRD and a set of hospital characteristics: hospital size, type (exclusively obstetrics or not), Neonatal Intensive Care Unit (NICU) availability, obstetrician-to-obstetric bed ratio, and teaching status.

**Results:**

CSR-LRD increased from 11.7% (2002) to 14.1% (2008), declining to 12.5% in 2011. While larger hospitals and hospitals with NICU had higher CSR-LRD rates, teaching status and obstetrician-to-obstetric bed ratio had no significant effect. Adjusted estimates, controlling for those four characteristics, indicate 91% of the variation in the CSR-LRD is left unexplained.

**Conclusion:**

Hospital characteristics do not explain variation in CSR-LRD rates. Further studies considering medical practice, financial incentives to hospitals and/or physicians, and patient education are needed.

## Highlights

• Focusing on c-section rates on low-risk deliveries minimises hospitals’ risk selection biases, e.g cream skimming.

• Hospital characteristics moderately affect c-section rates in low-risk deliveries.

• Policy changes should aim to promote established best practices rather than reducing available resources.

## Introduction

Caesarean section (CS) is the most frequently performed surgical procedure in the world [1, 2]. In 1985, the World Health Organization (WHO) issued general recommendations on CS, stating that the minimum acceptable rate was 1% and that there was no justification for any region to have rates higher than 10–15%. In 2015, the WHO once again stated that a CS rate (CSR) above 10% was not associated with reductions in maternal and new-born mortality rates [3] corroborated by Betran et al.’ systematic review of ecologic studies [4]. Plus, exposing mothers to unnecessary risks without additional benefits should only be undertaken when deemed medically necessary [2, 5].

Most high income countries exceeded the CSR threshold in 2002, up to an average of 21% of all births [6]. A study conducted in 24 countries and 373 health facilities in Africa, the Americas, and Asia observed that 25.7% of births were by CS, although rates varied significantly across countries suggesting that a number of CS were done without medical need [7].

In Portugal, policy makers have tried to curtail increasing CSR. Between 2004 and 2013, NHS hospitals’ rates increased from 27.9% to 30.8%, decreasing to 28.1% in the first nine months of 2014 [8], potentially due to maternal risk factors (RF). However, if that were the case, WHO’s CSR threshold would be too low [9]. Other non-clinical justifications for the increasing trends in CSR include obstetricians’ behaviour and medical practice [10–15], provider’s financial incentives, as well as the preferences or other time related incentives for mothers requesting elective CS [5, 16–19].

Regarding obstetrician incentives to perform CS over vaginal delivery, the most obvious incentive seems to be a financial one. Evidence showed that with the right financial incentives – similar payments for CS and vaginal delivery, for example – CSR might decrease [8, 20].

Opportunity costs of prolonged deliveries, minimization of malpractice risks or defensive medicine, and the demand for leisure or regular workweeks were also identified as reasons for the increasing numbers of CS [14, 21].

Differences in hospital CSR, after adjusting for maternal RF, have been attributed to pregnancy characteristics. Nevertheless, the increasing incidence of clinical indications for CS over time did not explain the rising rates [22]. Broad variation was found in preterm CS births [23] but differences in CSR could have resulted from almost random decision making [15, 20, 24]. Variation can also be due to uncertainty in diagnosis, although recent introduction of diagnostic technology may reduce hospital variation [25].

Importantly, availability of resources might increase CSR. Previous research has showed CSR increasing for admissions taking place from Monday to Thursday and decreasing for admissions on Friday and Saturday, when fewer staff was available [5]. In contrast, CS deliveries also increase resources due to longer lengths of stay, by two to three days, higher hospital costs and higher physician fees in comparison to vaginal deliveries [21, 26, 27].

At an aggregate level, supply factors played a critical role: the greater the hospital capacity of the health system, the greater the number of CS performed [28]. Moreover, obstetricians had a substantial influence on the delivery mode [28]. At the same time, hospital characteristics also seem to affect the CSR. It decreased when more specialized resources were available, including facilities with a neonatal intensive care unit (ICU) and maternal foetal medicine subspecialists [29]. The rates also decreased with the availability of obstetricians–gynaecologists specialists as opposed to family physicians only, higher delivery volume, urban location, and 24-hour in-house anaesthesiology. No differences were found between types of hospital ownership or by teaching status [5, 30, 31].

There is no evidence on the relationship between hospital characteristics and maternal risk profile, namely low-risk deliveries. This work aims to understand how hospital characteristics relate to c-section rates on low-risk deliveries, at the hospital level. By focusing our research on low-risk deliveries, biases related to hospitals’ case-mix and potential cream skimming are minimised and it singles out the effects of hospital resources on c-section rates.

Our study period is between 2002 and 2011. Due to changes in coding practices, data from more recent years would not be comparable with the data used in this work and the current percentage of c-sections observed in NHS hospitals is similar to one observed in 2010. Looking at data up to 2020, most recent data available, we can observe the following (Table 1):

**Table 1:** Number of deliveries (1,000) and percentage of C-sections in NHS hospitals, 2010-2020

Year	NHS deliveries (1,000)	C-sections (%)
2010	82.9	36.3
2011	77.6	35.8
2012	72.1	35.9
2013	66.1	35.6
2014	65.3	33.5
2015	67.7	32.9
2016	69.0	33.1
2017	68.6	33.1
2018	69.2	34.1
2019	69.2	36.0
2020	65.8	36.3

Source: PORDATA (www.pordata.pt)

The number of deliveries has been decreasing and CS were around 33% between 2015 and 2017, its lowest number. However, since 2018, an upward trend is being observed and we are now at the same values we had in 2010. This information strikingly shows that the situation has not changed much.

## Methods

Our database combined anonymized data on discharges in Portuguese NHS hospitals between 2002 and 2011 collected by the Central Administration of the Health System (Administração Central do Sistema de Saúde) and a dataset on the resources available at each NHS hospital for the same period collected by the Directorate General of Health (Direcção Geral de Saúde). Women aged between 15 and 55 years old grouped in Diagnosis Related Groups 370-373 (AP-DRG 21) were selected. To single out potential effects of risk factors from the analysis only low-risk deliveries were considered. The subset of low-risk deliveries was created by excluding all deliveries with registered conditions deemed as risk factors (RF) for CS (Table 2). The selection of conditions followed the definition of low-risk deliveries provided by García-Armesto et al. [32] When hospitals’ budgets are set based on DRGs, they are influenced by coding practices, therefore all hospitals in our dataset were faced with the same incentives to maximize their revenue. Furthermore, internal and external audits were carried out on a regular basis to assess the quality and accuracy of the coding standards [33].

**Table 2:** Risk factors – List of pregnancy characteristics and maternal medical conditions

Diagnosis Code (ICD-9-CM)	Description
641.11	Haemorrhage from placenta previa
641.21	Premature separation of placenta
641.31	Antepartum haemorrhage associated with coagulation defects
641.81	Other antepartum haemorrhage
641.91	Unspecified antepartum haemorrhage
642.01	Benign essential hypertension complicating pregnancy, childbirth, and the puerperium
642.51	Severe pre-eclampsia
642.61	Eclampsia
642.71	Pre-eclampsia or eclampsia superimposed on pre-existing hypertension
644.2x	Early onset of delivery
646.61	Infections of genitourinary tract in pregnancy
649.8x	Onset (spontaneous) of labour after 37 completed weeks of gestation but before 39 completed weeks gestation, with delivery by (planned) caesarean section
651.xx	Multiple gestation
652.2x	Breech presentation without mention of version
652.3x	Transverse or oblique presentation
652.4x	Face or brow presentation
652.60	Multiple gestation with malpresentation of one foetus or more
652.71	Prolapsed arm
654.01	Congenital abnormalities of uterus
654.11	Tumours of body of uterus
654.2x	Previous caesarean delivery
654.31	Retroverted and incarcerated gravid uterus
654.41	Other abnormalities in shape or position of gravid uterus and of neighbouring structures
654.51	Cervical incompetence
654.61	Other congenital or acquired abnormality of cervix
654.71	Congenital or acquired abnormality of vagina
656.31	Foetal distress
656.4x	Intrauterine death
656.81	Other specified foetal and placental problems
658.11	Premature rupture of membranes
658.21	Delayed delivery after spontaneous or unspecified rupture of membranes
659.01	Failed mechanical induction
659.11	Failed medical or unspecified induction
659.31	Generalized infection during labour
660.x1	Obstructed labour
662.3x	Delayed delivery of second twin, triplet, etc.
663.01	Prolapse of cord
663.11	Cord around neck, with compression
663.21	Other and unspecified cord entanglement, with compression
665.01	Rupture of uterus before onset of labour
665.11	Rupture of uterus during labour
665.31	Laceration of cervix
668.1x	Cardiac complications
669.01	Maternal distress
669.11	Shock during or following labour and delivery
669.61	Breech extraction, without mention of indication
042	Human immunodeficiency virus [HIV] disease

A C-section rate in low-risk deliveries (CSR-LR) was calculated for each hospital in each year.

Hospital characteristics considered in this analysis included: hospital size (number of beds per hospital), hospital specialization in obstetrics (availability of neonatal ICU, binary variable: yes/no), availability of obstetric resources (ratio of obstetricians to obstetric beds) and teaching status (binary variable: yes/no). Summary statistics can be found in Table 3.

**Table 3:** NHS hospitals summary statistics for number of beds, availability of NICU, obstetric resources, and teaching status, 2002-2011

	2002	2003	2004	2005	2006	2007	2008	2009	2010	2011
**Number of hospitals**	50	50	50	50	49	40	40	40	40	39
**Hospital dimension (number of beds per hospital)**										
Average	381	381	381	382	398	505	505	508	508	592
Standard deviation	274	278	279	277	281	299	305	304	297	382
Minimum	97	97	97	97	97	143	143	138	139	126
Maximum	1,525	1,530	1,548	1,505	1,497	1,496	1,456	1,456	1,375	2,070
**Hospital specialization in obstetrics (availability of NICU: yes/no)**										
% of hospitals (yes)	28%	32%	32%	30%	37%	40%	40%	45%	45%	36%
**Availability of obstetric resources (ratio of obstetricians to obstetric beds)**										
Average	0.363	0.344	0.347	0.333	0.337	0.359	0.371	0.364	0.388	0.375
Standard deviation	0.144	0.120	0.123	0.118	0.122	0.130	0.149	0.147	0.200	0.160
Minimum	0.105	0.154	0.125	0.000	0.053	0.068	0.125	0.098	0.125	0.125
Maximum	0.857	0.857	0.886	0.686	0.657	0.686	0.755	0.819	1.191	0.864
**Teaching status (teaching hospital: yes/no)**										
% of hospitals (yes)	92%	94%	94%	98%	98%	98%	98%	98%	95%	95%

*NICU* Neonatal Intensive Care Unit

Source: *Direcção Geral de Saúde*

The reduction in the number of hospitals between 2002 and 2011 was due to hospital mergers taking place over the period. That organizational fact influenced not only the number of institutions analysed in each year but also the resources of each hospital which were combined as a new institution was created. As a consequence, the average number of beds available at hospitals increased over the years as well as the percentage of hospitals with a neonatal ICU and the number of teaching hospitals. No more than three hospitals nationwide had no teaching activities, nevertheless, it was decided that the variable should be controlled for to understand whether the differences appeared to be systematic.

Fractional Response Models (FRM) are used when the response variable is a proportion and is bounded between 0 and 1. These models accommodate this boundness and avoid estimation of nonsensical predictions (out of bounds of the standard unit interval) by assuming that the effects of explanatory variables are not linear (not constant through all of its range as the variance tends to decrease when the mean gets closer to the boundaries) [34, 35]. Both cross-sectional and panel data models were estimated in the present study. FRMs (cross-sectional and panel data) required the correct specification of the conditional expectation of the fractional response variable, i.e., a functional form for the distribution of the CSR-LRD must be assumed. This functional form imposed constraints on the conditional mean of this variable. Frequent choices for the functional form include logistic, probit, loglog, cloglog and cauchit functions. Details about these functions may be found in Ramalho et al. [35]. A cloglog distribution was selected for the functional form (both for cross-sectional and panel data) as CSR-LRD tended to be closer to 0. In the panel data model, the pooled fixed-effects estimator described in Ramalho et al. [36] was used. This estimator was chosen because it captured individual hospital characteristics that may not have been reflected in the explanatory variables related to hospital characteristics, but that could have been correlated with this set of variables. In both cross-sectional and panel data models, year-dummies were included among the explanatory variables.

Ordinary Least Squares (OLS) and Fixed-Effects linear panel data model (PLM-FE) were also estimated for comparison. R2 were studied when available. For the cross-sectional estimation of the FRM, RESET-type tests and the generalised goodness-of-functional form (GGOFF) tests were performed. Both tested if the functional form assumed corresponded to the conditional mean and could be interpreted as a test for the omission of other explanatory variables in the model. If the functional form was correctly specified, the null hypothesis should be accepted. The choice of the cloglog for the functional form turned out to be supported by specification tests GGOFF and RESET. There were no tests available for panel data estimation in FRM.

SPSS and R were used for the summary statistics and econometric model estimation.

## Results

Table 4 presents general information of the evolution of deliveries, low-risk deliveries, CSR-LRD and CSR-LRD at hospital level. Deliveries were continuously decreasing until 2010 when a slight increase occurred. CSR steadily increased from 26% in 2002 to 31.6% in 2009, when the rate started to slightly decline to 29% in 2011. These values were still much higher than the widely quoted 15% threshold recommended by the WHO.

**Table 4:** Summary statistics on Portuguese NHS hospitals deliveries, low risk deliveries, c-sections, c-section low risk deliveries and c-section rate in low-risk deliveries, 2002-2011

Year	No Hospitals	All Deliveries DRGs 370-373 [a]	Low-Risk Deliveries (% of [a]) [b]	C-Sections All Deliveries (CSR, % of [a])	C-Sections Low-Risk Deliveries (CSR-LR, % of [b])	C-Section Rate in Low-Risk Deliveries (Hospital level)		C.V. *σ*/*μ*
						Mean (S.D.) μ (σ)	Min – Max	
2002	50	93,649	54,533 (58.2%)	24,357 (26.0%)	6,372 (11.7%)	12.4 (8.1)	2.0 – 45.0	0.653
2003	50	90,888	52,901 (58.2%)	25,108 (27.6%)	6,502 (12.3%)	13.2 (7.9)	2.1 – 45.8	0.598
2004	50	88,086	51,419 (58.4%)	25,101 (28.5%)	6,565 (12.8%)	13.6 (7.4)	2.7 – 34.2	0.544
2005	50	87,701	49,597 (56.6%)	26,334 (30.1%)	6,542 (13.2%)	14.2 (7.8)	2.8 – 37.2	0.549
2006	49	85,023	47,365 (55.7%)	25,884 (30.5%)	6,407 (13.5%)	14.4 (8.0)	1.3 – 40.4	0.556
2007	40	82,309	44,796 (54.4%)	25,515 (31.0%)	6,217 (13.9%)	13.7 (8.0)	2.4 – 31.5	0.584
2008	40	82,696	44,497 (53.8%)	26,097 (31.6%)	6,266 (14.1%)	13.9 (7.8)	1.0 – 30.4	0.561
2009	40	78,752	41,004 (52.1%)	24,884 (31.6%)	5,573 (13.6%)	13.4 (7.2)	2.0 – 30.2	0.537
2010	40	71,058	37,161 (52.3%)	21,561 (30.3%)	4,967 (13.4%)	12.9 (6.5)	1.9 – 28.3	0.504
2011	39	73,963	38,712 (52.3%)	21,540 (29.1%)	4,824 (12.5%)	12.4 (5.6)	1.1 – 24.6	0.452

Note: DRG – Diagnosis Related Group; S.D. – Standard Deviation; C.V. – Coefficient of Variation; μ – mean; σ – standard deviation

Low-risk deliveries accounted for 55.4% of total deliveries and followed the same tendency decreasing in total until 2010 and slightly increasing in 2011. CSR-LRD were much lower than CSR as was anticipated. The trend nevertheless was similar; CSR-LRD increased between 2002 and 2008 from 11.7% to 14.1% and then decreased to 12.5% in 2011.

At hospital level, CSR-LRD varied from 1% to 45.8%. The coefficient of variation ranged from 45.2% to 65.3% over the years of analysis presenting a large dispersion of CSR-LR across hospitals.

### Linear vs Fractional response models

Model estimations are presented in Table 5. The sign and statistical significance of cross sectional estimates were consistent across linear and FRM. For panel data, this statement was true for hospital characteristics except “teaching” but not true for year-dummies which assumed some significance in the linear model but not on the FRM model.

**Table 5:** Model estimations for C-section rates in low-risk deliveries: Fractional response models and linear models

C-Section Rates in	Fractional Response Models				Linear Models			
Low-Risk Deliveries	(Functional Form: Cloglog distribution)							
*N*=442			Panel-Data		Cross-Sectional		Panel-Data	
*n*=70; T=1-10	Cross-Sectional		(Pooled Fixed-Effects)		(Ordinary Least Squares)		(Fixed-Effects)	
	Coefficient	(SE) Sig. Level	Coefficient	(SE) Sig. Level	Coefficient	(SE) Sig. Level	Coefficient	(SE) Sig. Level
Intercept	-1.769	(0.139)***	-	0.161	(0.020)***	-		
Number of beds	0.016	(0.009)*	-0.023	(1.178)	0.002	(0.001)°	-0.007	(0.007)
Availability of Neonatal ICU	0.251	(0.054)***	0.029	(0.237)	0.032	(0.008)***	0.005	(0.008)
Obstetricians per obstetric beds	0.122	(0.166)	-0.248	(1.953)	0.013	(0.025)	-0.033	(0.024)
Teaching hospital	-0.477	(0.126)***	0.311	(1.227)	-0.065	(0.017)***	0.044	(0.016)**
Year 2003	0.068	(0.131)	0.058	(0.181)	0.008	(0.015)	0.006	(0.008)
Year 2004	0.092	(0.130)	0.099	(0.206)	0.012	(0.015)	0.009	(0.008)
Year 2005	0.173	(0.129)	0.126	(0.167)	0.022	(0.015)	0.013	(0.008)°
Year 2006	0.169	(0.130)	0.118	(0.235)	0.021	(0.015)	0.015	(0.008)°
Year 2007	0.088	(0.134)	0.155	(0.261)	0.010	(0.016)	0.018	(0.008)*
Year 2008	0.097	(0.131)	0.177	(0.356)	0.012	(0.016)	0.022	(0.009)*
Year 2009	0.049	(0.128)	0.166	(0.388)	0.005	(0.016)	0.017	(0.009)°
Year 2010	-0.008	(0.127)	0.183	(0.359)	-0.001	(0.016)	0.014	(0.009)
Year 2011	-0.040	(0.126)	0.195	(0.404)	-0.005	(0.016)	0.014	(0.009)
**R**^**2**^	**0.086**				**0.088**		**0.066**	
**Reset test (p-value)**								
LM(2)	0.558 (0.455)							
LM(3)	1.167 (0.558)							
**Goodness of Functional Form test**								
**(p-value)**								
GOFF1 (LM)	0.605 (0.437)							
GGOFF (LM)	0.605 (0.437)							

Robust Standard Errors; *ICU* Intensive Care Unit, *LM* Lagrange Multiplier, *GOFF* Goodness of functional form, *GGOFF* Generalised goodness of functional form

Sig. Level: ***p-value<0.001; ** *p*-value<0.01; *p-value < 0.05; *p*-value <0.1.

For cross-sectional FRM estimates, GGOFF and RESET tests were not rejected indicating that the cloglog functional form was adequately selected to estimate the models. These tests were not available for panel data FRM*.* Nevertheless, standard errors of the parameters from linear models were smaller than those from FRMs. Using linear models to predict fractional response variables may be more efficient when the analysis is not concentrated on extreme values of 0 and 1 as happened in this situation.

### Cross-Sectional vs Panel data analysis

Although R^2^ was higher for cross-sectional models, in both linear and FRM when panel data models were estimated hospital characteristics lost significance and even reversed sign. In the panel data of linear model, some year-dummies were significant. This suggested that hospital previous results, past practice and year may have been more relevant than the hospital characteristics considered in the analysis.

### Hospital characteristics

Availability of Neonatal ICU was the only variable with consistent positive effect on CSR-LRD across the four models, although the estimates were significant only in cross-sectional models. In the cross-sectional models availability of Neonatal ICU was significant and the rate of CSR-LRD significantly increased with number of beds per hospital.

Teaching hospitals were associated with lower levels of CSR-LRD in cross-sectional models and these results were statistically significant. However, when extending the analysis to a panel data model this relation became positive.

The effect of ratio of obstetricians to obstetric beds was not significant in any model, being positive for cross-sectional and negative for panel data models.

For all the models estimated, the *R*^2^ was small and thus, the selected hospital characteristics and year only explain a small part of the variations observed in the CSR-LRD of hospitals.

## Discussion

In this study, we aim to understand the relationship between hospital characteristics and c-section rates, focusing on low-risk deliveries, at the hospital level. This approach is especially relevant when working at hospital level since it minimises biases related to hospitals’ case-mix.

So far, the literature has justified the variability in CSR with differences in medical practice, physicians’ behaviours, patients’ risk profile or preferences, hospitals’ characteristics and availability of resources [10–13, 15, 16, 20, 22–29]. Availability of resources has been suggested to increase activity regardless of its need, but specialized resources have been linked to lower clinically unnecessary CSR [29].

Our results were aligned with the literature. The availability of specialised resources is linked to higher CSR, measured by the positive correlation between neonatal ICU and number of beds with CSR-LRD.

When other variables were considered in the models, the ratio of obstetricians to obstetric beds was not significant suggesting that the relationship between obstetric resources availability did not explain the differences found in CSR-LRD.

When panel data models were estimated, accommodating for time fixed effects, hospital characteristics became not significant. This suggested that, apart from hospital characteristics, there may have been other factors explaining the differences between the CSR-LRD at hospital level. Moreover, even when statistically significant, marginal effects of hospital characteristics were small and changes in resources would only affect CSR-LRD slightly. The *R*^*2*^ was small conveying that the selected hospital characteristics were not very relevant in justifying variations across hospitals.

Auditing and feedback on all CS, second opinions, training of doctors on caesarean delivery guidelines and implementation of health education and behaviour change strategies, have all been hailed as crucial measures to reduce CSR, without affecting patient outcomes. However, the literature is inconclusive. Some authors have found positive results [37–39], while others have found modest results [40–42]. The findings of Epstein and Nicholson [41], for example, suggested that experience sharing did not lead to physicians’ prior belief or practice change. Other suggestions for reducing rates include annual publication of the CSR per hospital and the inpatient rate due to hypoxic-ischemic new-borns, equalizing the payment of CS and vaginal delivery, financial incentives to hospitals that present lower CSR, implementation of an operating theatre next to a delivery room and the implementation of a rule of non-induction of labour with no medical reason before 41 weeks gestation [8]. In Portugal, the implementation of these measures seems to have led to a decrease in NHS hospitals CSR, to 28.1%, in 2014 [8].

There were some discrepancies between the results found in this work and the results from other authors. This work focus was on low-risk deliveries as opposed to using the general CSR or the common nulliparous, term, singleton and vertex births (NTSV) and thus results were not truly comparable. Nevertheless, CSR did not account for CS that were clinical justifiable and the NTSV definition has proven not to be sufficient to eliminate potential clinical justification for CS. This lack of comparison also happened when using other metrics and comparing results across countries since there have been international discrepancies in the classification of deliveries without complications [43].

### Strengths and limitations

This paper has an innovative modelling approach. So far different models were used in the literature with some authors modelling the probability of CS delivery and not CSR-LRD at hospital level. The choice of FRM to model the research question was technically the most appropriate given the fractionary nature of the response variable. As for the choice of the panel-data model, this estimator could accommodate individual characteristics of hospitals beyond hospital characteristics already included.

The small explanatory power of the models revealed that much of the variation in the CSR-LR was still to be explained, but further variables relating to medical practice and organizational settings were not available, as weren’t mothers’ preferences or economic incentives to the physicians. However, at the time of the analysis, economic incentives to change physicians’ behaviours had not been implemented in Portugal therefore their impact could not have been assessed.

Additional limitations relate to aggregated information on merged hospitals which made us lose additional insights on individual hospitals, data unavailability of other potential explanatory variables such as anaesthesiologists who are required to perform CS and the role of hospitals’ financial incentives on performance.

## Conclusion

Policies aiming at reducing CSR, namely CSR-LRD, should not focus on increasing efficiency by reducing available resources. In fact, policies targeting practice change may be more effective, as reported in literature [37–39]. This can be achieved not only by introducing economic incentives, but also by investing in continuous training of doctors, through the development of shared obstetric protocols and monitoring adherence to standardized clinical guidelines, and by promoting trial of labour after caesarean (TOLAC) where appropriate. Possible interventions include performing peer reviews, obtaining second opinions on adequacy for all CS, and auditing representative samples of CS cases, as suggested by different authors [8, 15, 20, 37–39]. Further, raising awareness of staff/patients by opinion leaders and prenatal counselling for women and partners would be important. Public health campaigns aimed at the general population on the risks associated with CS could also be given a careful thought. We expect that our findings contribute to policy makers designing effective policies to reduce c-section rates at the hospital and national level.

## Data Availability

Data is not available to be shared with other researchers.
